# Genetic prediction of the relationship between metabolic syndrome and colorectal cancer risk: a Mendelian randomization study

**DOI:** 10.1186/s13098-024-01351-7

**Published:** 2024-05-22

**Authors:** Chendong Yuan, Xufeng Shu, Zhenzhen Hu, Zhigang Jie

**Affiliations:** 1 Department of General Surgery, The First Affiliated Hospital, Jiangxi Medical College, Nanchang University, Nanchang, 330006 Jiangxi China; 2 Medical Innovation Center, The First Affiliated Hospital, Jiangxi Medical College, Nanchang University, Nanchang, 330006 Jiangxi China; 3 Department of Anesthesiology, The First Affiliated Hospital, Jiangxi Medical College, Nanchang University, Nanchang, 330006 Jiangxi China

**Keywords:** Metabolic syndrome, Colorectal cancer, Abdominal obesity, Mendelian randomization, Waist circumference

## Abstract

**Background:**

Despite a growing body of observational studies indicating a potential link between metabolic syndrome and colorectal cancer, a definitive causal relationship has yet to be established. This study aimed to elucidate the causal relationship between metabolic syndrome and colorectal cancer through Mendelian randomization.

**Methods:**

We screened for instrumental variables associated with metabolic syndrome and its diagnostic components and with colorectal cancer through the use of a genome-wide association study database, and conducted a preliminary Mendelian randomization analysis. To corroborate the dependability of our conclusions, an additional dataset was used for replication analysis in a Mendelian randomization method, which was further integrated with a meta-analysis.

**Results:**

Preliminary analysis using the inverse variance weighted method revealed positive correlations between metabolic syndrome (OR [95% CI] = 1.37[1.15–1.63], *P* = 5.02 × 10^–4^) and waist circumference (OR [95% CI] = 1.39[1.21–1.61], *P* = 7.38 × 10^–6^) and the risk of colorectal cancer. Replication analysis also revealed the same results: metabolic syndrome (OR [95% CI] = 1.24[1.02–1.51], *P* = 0.030) and waist circumference (OR [95% CI] = 1.23[1.05–1.45], *P* = 0.013). The meta-analysis results further confirmed the associations between metabolic syndrome (OR [95% CI] = 1.31[1.15–1.49], *P* < 0.001) and waist circumference (OR [95% CI] = 1.32[1.18–1.47], *P* < 0.001) and colorectal cancer.

**Conclusion:**

Our study indicated that metabolic syndrome increases the risk of CRC, particularly in patients with abdominal obesity.

**Supplementary Information:**

The online version contains supplementary material available at 10.1186/s13098-024-01351-7.

## Introduction

Colorectal cancer (CRC) is currently the third most prevalent malignancy and the second most deadly malignancy worldwide. Annually, CRC accounts for an estimated 1.8 million new cases and 880,000 fatalities, representing approximately 10% of the total cancer incidence and mortality rates [1, 2]. The CRC is considered an indicator of increased human development indices [3]. In recent years, as quality of life has improved and dietary habits have shifted, the incidence of CRC has steadily increased. This trend may be attributed to factors such as unhealthy diets, sedentary lifestyles, and smoking, which are often accompanied by overweight or obesity and are closely related to metabolic syndrome (MetS) [4].

MetS, as defined by the International Diabetes Federation, is characterized by the presence of a minimum of three out of five diagnostic components: enlarged waist circumference (WC), high diastolic blood pressure (DBP) or systolic blood pressure (SBP), increased triglycerides (TG), low HDL cholesterol (HDL-C), and elevated fasting glucose (FG) [5]. The global prevalence of MetS is on the rise, with estimates suggesting that approximately one quarter of the global population fulfils the diagnostic criteria for MetS. MetS is considered to be a significant factor influencing cancer incidence, and in recent years, its relationship with CRC has attracted widespread attention [6, 7]. Previous studies have shown that MetS not only affects the incidence of CRC but also influences its progression and has even become an independent risk factor for the metastasis and recurrence of CRC [8–10]. Additionally, many studies have indicated that the diagnostic components of MetS are linked to a heightened risk of CRC [11]. However, since most of the current evidence is derived from observational studies, these studies are susceptible to confounding factors, such as small sample sizes, selection bias, and limited follow-up duration, which could lead to biased results. Therefore, it is difficult to determine whether MetS is causally related to CRC.

Mendelian randomization (MR) represents a methodology employed to evaluate causal associations between exposures and outcomes, utilizing single nucleotide polymorphisms (SNPs) linked with the exposure as instrumental variables (IVs) [12]. The random allocation of SNPs to offspring at conception substantially mitigates bias arising from confounding factors [13]. Genome-wide association study (GWAS) have identified a multitude of SNPs associated with CRC and MetS, including their diagnostic components. Consequently, this study utilizes MR analysis to explore the causal relationship between them.

## Materials and methods

### Study design

In the MR study, MetS and its diagnostic components were considered as exposure. SNPs related to these exposures were selected as genetic IVs and were sourced from publicly accessible GWAS datasets. It was employed to assess the causal relationship between exposure and outcome (e.g., CRC). The selected SNPs, serving as effective genetic IVs, must satisfy three core assumptions of MR [14]: (1) a robust association is observed between the selected genetic IVs and the exposure; (2) the genetic IVs are independent of any confounders that may affect the relationship between the exposure and the outcome; and (3) the influence of the genetic IVs on the outcome is exerted exclusively through the exposure.

### GWAS data resources

The GWAS data on MetS were sourced from the CNCR database (https://ctg.cncr.nl/). van Walree et al. [15], selectively included individuals of European ancestry, ultimately using an effective population size of 461,920, and identified 235 genomic risk loci correlated with MetS. Genetic data on FG were obtained from the MAGIC (https://magicinvestigators.org/). Scott et al. [16], conducted a genome-wide association meta-analysis of 133,010 individuals of European ancestry without diabetes and identified 64,432 associated SNPs. The GWAS ATLAS (https://atlas.ctglab.nl/) was the source of the genetic dataset related to SBP. Watanabe et al. [17], aggregated data from 361,402 individuals of European ancestry and identified 257 independent genomic risk loci and 10,534,620 SNPs. The GWAS dataset for TG was released from the Center for Statistical Genetics (https://csg.sph.umich.edu/). Teslovich et al. [18], published a study in which a total sample size of 96,598 individuals of European ancestry was included, and 2,625,646 SNPs were screened. Genetic datasets for WC, DBP, and HDL-C were all released through the IEU Open GWAS project (http://gwas.mrcieu.ac.uk/). The datasets for WC and DBP originated from MRC-IEU, which involved statistical analysis of 462,166 and 436,424 individuals of European ancestry, respectively, and analyzed 9,851,867 SNPs in total. The dataset for HDL-C was disclosed in research by Howe LJ et al [19]. This research amalgamated data from 178,086 siblings spanning 19 distinct cohorts, culminating in the derivation of GWAS estimates for 25 phenotypes, inclusive of HDL-C, sample size of 37,120 (Table 1).

**Table 1:** Overview of GWAS data related to exposure

Exposure (ICD)	Ancestry	Simple size	Author	Institution	PMID
Metabolic syndrome (E88.81)	European	461,920	van Walree et al	CNCR database	35983957
Fasting glucose	European	133,010	Scott et al	MAGIC	22885924
HDL cholesterol	European	37,120	Howe LJ et al	Within family GWAS consortium	35534559
Triglycerides	European	96,598	Teslovich et al	Center for Statistical Genetics	20686565
Systolic blood pressure	European	361,402	Watanabe et al	GWAS ATLAS	31427789
Diastolic blood pressure	European	436,424	Ben Elsworth et al	MRC-IEU	/
Waist circumference	European	462,166	Ben Elsworth et al	MRC-IEU	/

*ICD* international classification of diseases

Summary data for CRC from GWAS were obtained from the GWAS Catalog (http://www.ebi.ac.uk/gwas) using the accession number GCST012876. This dataset, encompassing 38,370,461 SNPs, originated from a study conducted by Huyghe et al [20]. The study's cohort consisted of 26,554 individuals of European ancestry, comprising 11,895 cases and 14,659 controls. This CRC dataset was utilized for preliminary analysis. All the data samples in the abovementioned studies included both male and female individuals.

### IVs selection

To adhere to assumption 1, we identified IVs associated with exposure through stringent conditions. Initially, we selected genetic variant SNPs with genome-wide significance (*P* < 5 × 10^–8^) that were significantly associated with exposure to serve as IVs. Subsequently, to ensure the independence of each SNP, we employed the PLINK algorithm (parameters: r^2^ < 0.001, clumping window = 10,000 kb) to clump SNPs, eliminating linkage disequilibrium. Furthermore, to address potential biases arising from weak IVs, the F-statistics for each SNP were calculated using the formula F-statistics = β^2^/SE^2^, where β represents the effect size of exposure IVs, and SE represents the standard error of β [21]. If the value of F statistics was less than 10, identifying the variable as a weak IV was excluded [22]. Next, we extracted SNPs significantly associated with the exposure (*P* < 5 × 10^–8^) and further harmonized the SN*P*s for exposure and outcome, also eliminating palindromic SNPs, because these can introduce potential linkage flip issues, leading to ambiguity in the allele-specific coordination of effects between the exposure and outcome. Finally, the remaining SNPs were subjected to MR analysis.

### MR analysis and sensitivity analysis

In this study, we estimated the causal relationship between MetS, its diagnostic components, and CRC using five distinct MR methods which included the inverse variance weighted (IVW), MR‒Egger, weighted median, simple mode, and weighted mode methods. IVW aggregates the Wald ratio of each SNP, and under the assumption of valid IVs, it can provide the most accurate estimates [23]. If genetic IVs affect the outcome through pathways other than exposure, the IVW method may identify biased results. Therefore, the MR-Egger method can serve as a supplement to IVW, introducing an intercept term in the regression model to adjust for biases in genetic IVs, thereby yielding more stable conclusions [24]. As long as more than 50% of the IVs are valid, the weight median can offer accurate causal effect estimations [25]. The simple mode is particularly affected by the heterogeneity of IVs. This method selects the mode of the distribution of causal estimates, providing robustness against a subset of invalid IVs. On the other hand, weighted mode improves upon simple mode by weighting each IVs' estimate by its inverse variance, making it ideal for datasets where IVs vary significantly in their reliability. This method is beneficial when some pleiotropy is present. In brief, simple mode and weighted mode can provide better casual estimates when dealing with instrumental variables of varying reliability, yet their effectiveness remains limited [26]. Overall, we primarily assess the causal relationship between MetS and its diagnostic components and CRC using the IVW method, with the other four MR methods serving as supplementary analyses. The causal effect estimations and confidence intervals of the five methods are displayed in a forest plot.

Furthermore, to ascertain the robustness of our findings, several sensitivity analyses were conducted. These included Cochran’s Q test, the MR‒Egger intercept test, MR-PRESSO, leave-one-out (LOO) analysis, and funnel plots. Cochran’s Q test was used to evaluate heterogeneity among the IVs; significant heterogeneity was indicated by a *P* value of less than 0.05, necessitating the adoption of a random-effects IVW model over a fixed-effect IVW model [27]. We calculated the MR-Egger intercept to evaluate the potential pleiotropic effects exerted by the IVs, where a *P* value less than 0.05 indicated the likelihood of horizontal pleiotropy [24]. Subsequently, we used the MR-PRESSO global test, which provides a higher level of evidence, to further evaluate horizontal pleiotropy. Additionally, the MR-PRESSO outlier and distortion test was applied to identify and adjust for any outliers, thereby correcting for horizontal pleiotropy by excluding outlier SNPs, thus fulfilling assumption 2 of MR [28]. LOO analysis was conducted by sequentially removing each SNP associated with the exposure and repeating the IVW analysis to evaluate whether the results were heavily influenced by individual SNPs [29]. A funnel plot was used to assess the robustness of the results.

### Replication and meta-analysis

To evaluate the reliability of the causal relationship between MetS and its diagnostic components with CRC, we replicated the MR and sensitivity analyses in another CRC cohort. The datasets for this cohort were also sourced from the GWAS Catalog, with the GWAS accession number GCST012877. This GWAS dataset included a total sample of 23,691 individuals of European ancestry, including 11,835 cases and 11,856 controls, and identified a total of 12,313,483 SNPs. In brief, GWAS datasets bearing the accession number GSCT012876 were utilized for preliminary analysis. For the replication analysis, GWAS datasets with the accession number GCST012877 were used. The causal relationship between MetS and its diagnostic components in different CRC datasets were combined with meta-analyses, which quantified the heterogeneity between different dataset estimates using the I^2^ statistic and corresponding *P* value from Cochran’s Q test. In instances where significant heterogeneity was observed (I^2^ > 50% and *P* < 0.05), a random-effects model was used for the meta-analysis. Conversely, in the absence of significant heterogeneity, a fixed-effects model was applied.

### Statistical power and genetic directionality assessment

Additionally, when the direction of causality or potential pleiotropy is unclear, genetic variations affecting multiple traits may influence outcomes through pathways other than exposure. Therefore, the Steiger test was used to satisfy assumption 3 of MR and refute reverse causality, ensuring that the genetic IVs primarily affect exposure and not directly the outcome [30].

In summary, during the MR analysis process, we identified MetS and its diagnostic components having potential causal effects on CRC through strict criteria: (1) IVW *P* < 0.05 and meta-analysis* P* < 0.05. (2) The direction of the five MR methods is consistent, i.e., the odds ratios (OR) are either greater than 1 or less than 1. (3) After removing outlier SNPs, the MR results show no heterogeneity or horizontal pleiotropy. (4) MR estimates are not significantly affected by individual SNPs. (5) There is no reverse causality. To ensure that MR studies have sufficient statistical power to detect the estimation of the causal effects of IVs on the exposure and outcome, we calculated statistical power using an online website (https://sb452.shinyapps.io/power/) [31]. Overall, we set the Type I error rate at 0.05 and calculated the statistical power using the outcome's sample size, case odds ratio, the proportion of variance explained (PVE) of selected IVs, and the OR from the IVW method. When the statistical power exceeds 80%, we consider the MR analysis results to be highly reliable.

MR analysis was conducted using the 'TwoSampleMR' and 'MR-PRESSO' packages in R software version 4.3.1, and meta-analysis was conducted using the 'meta' package. The data utilized in our research were exclusively sourced from publicly available genetic databases. Consequently, the necessity of obtaining ethical approval from the affiliated ethics committees of the authors was waived.

## Results

### Preliminary analysis

**Fig. 1:**
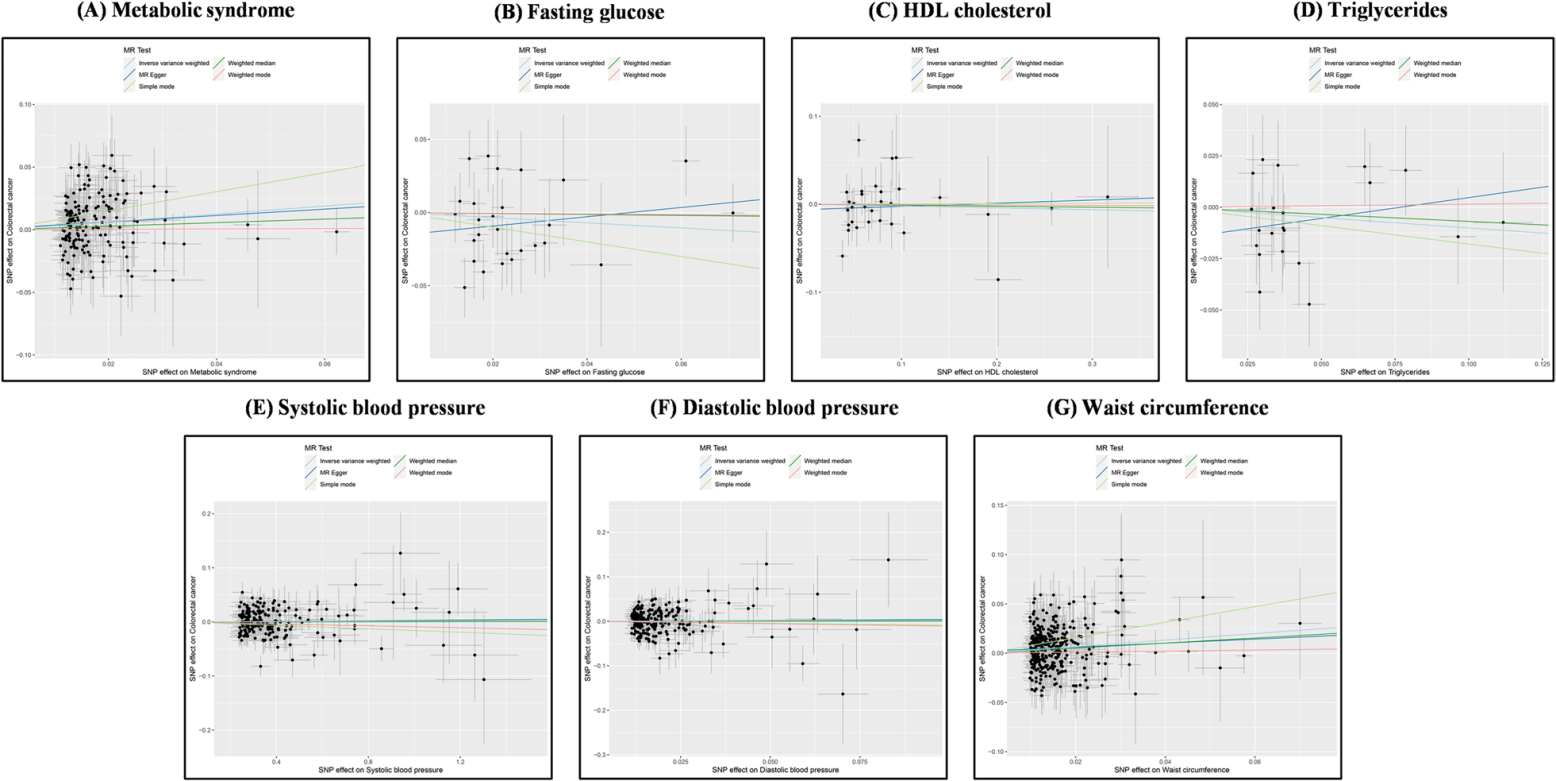
In the preliminary analysis, scatter plot demonstrated the impact of MetS and its diagnostic components on CRC. **A** Scatter plot of metabolic syndrome and colorectal cancer; **B** Scatter plot of fasting glucose and colorectal cancer; **C** Scatter plot of HDL cholesterol and colorectal cancer; **D** Scatter plot of Triglycerides and colorectal cancer; **E** Scatter plot of systolic blood pressure and colorectal cancer; **F** Scatter plot of diastolic blood pressure and colorectal cancer; **G** Scatter plot of waist circumference and colorectal cancer. *SNP* single nucleotide polymorphism, *MR* Mendelian randomization

**Fig. 2:**
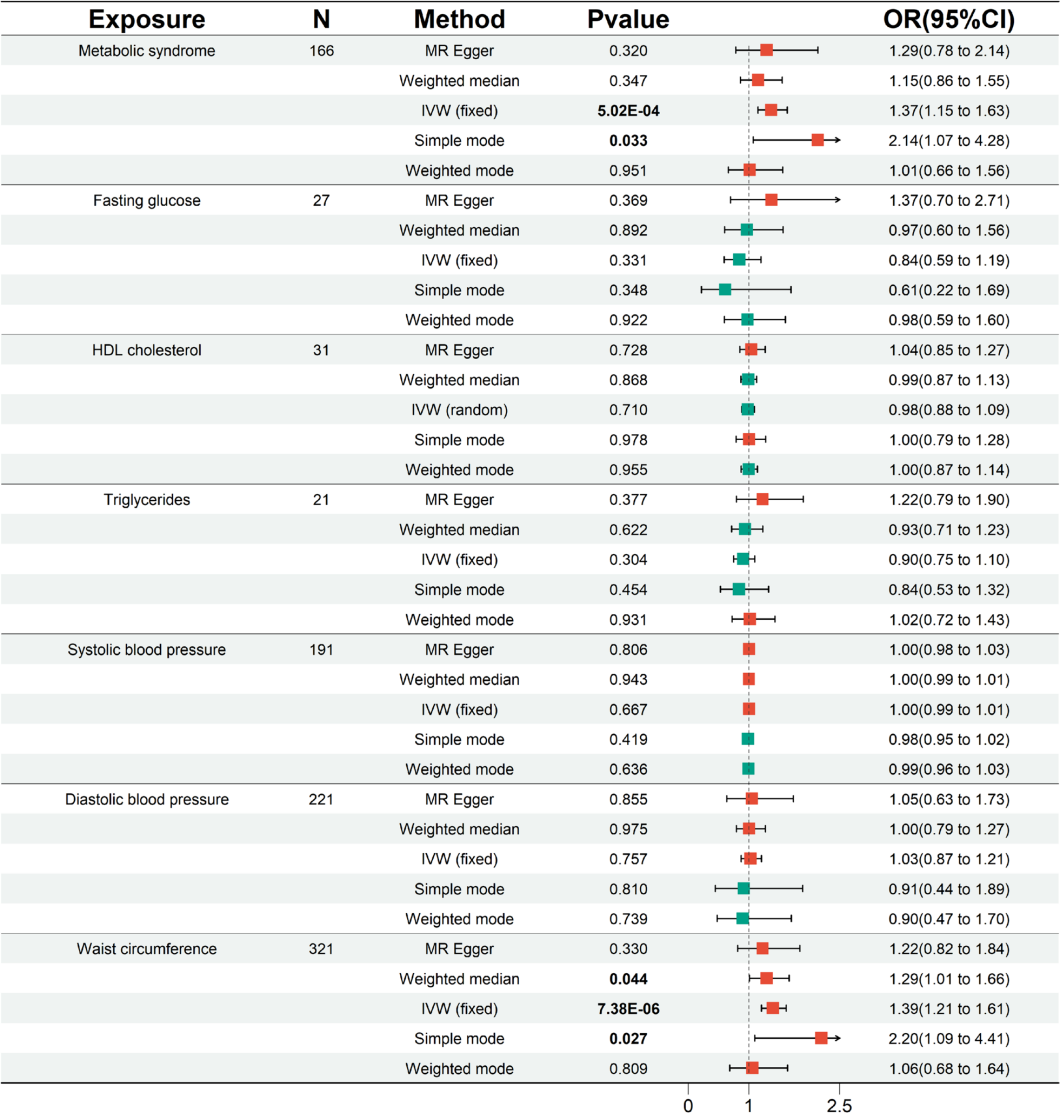
In the preliminary analysis, forest plot for metabolic syndrome and its diagnostic components. *95% CI* 95% confidence interval, *OR* odds ratio, *N* the number of SNPs strongly associated with the exposure, *IVW* inverse variance weighted

We used the PLINK algorithm to clump SNPs, excluding palindromic SNPs and weak SNPs (F statistic < 10). However, in the subsequent MR-PRESSO global test, we detected horizontal pleiotropy in the FG (*P* < 0.001), TG (*P* = 0.003), and DBP (*P* = 0.016). Through the MR-PRESSO outlier and distortion test, we recognized and removed all outliers in the FG (*P* = 0.574; outliers: rs1260326, rs174576, rs2191349), TG (*P* = 0.707; outliers: rs12678919, rs174546, rs1260326), and DBP (*P* = 0.122; outliers: rs10774625, rs41475048). Ultimately, we identified SNPs strongly associated with MetS (N = 166), FG (N = 27), HDL-C (N = 31), TG (N = 21), SBP (N = 191), DBP (N = 221), and WC (N = 321) as genetic IVs. Subsequently, a preliminary MR analysis was conducted (Figs. 1 and 2). The IVW results (OR [95% CI] = 1.37[1.15–1.63], *P* = 5.02 × 10^–4^, statistical power = 99.3%) revealed a significant association between genetic susceptibility to MetS and increased CRC risk. This association was further confirmed by simple mode analysis (OR [95% CI] = 2.14[1.07–4.28], *P* = 0.033). Concurrently, the IVW (OR [95% CI] = 1.39[1.21–1.61], *P* = 7.38 × 10^–6^, statistical power = 100%), weight median (OR [95% CI] = 1.29[1.01–1.66], *P* = 0.044), and simple mode (OR [95% CI] = 2.20[1.09–4.41], *P* = 0.027) all indicated statistically significant and directionally consistent results, suggesting that an increase in WC raises the risk of CRC. According to the MR analyses of MetS and WC, the direction and magnitude of the estimates from all five MR methods were consistent (OR > 1). The remaining diagnostic components of MetS (e.g., FG, HDL-C, DBP, SBP, and TG) showed no statistically significant association with CRC risk (IVW: *P* > 0.05), however, it is noteworthy that their calculated statistical power values are all less than 80%, with FG at 51.5%, HDL-C at 7.1%, TG at 22.6%, SBP at 2.5%, and DBP at 6.1%. Furthermore, Cochran’s Q test indicated heterogeneity in the HDL-C results (MR‒Egger: *P* = 0.040, IVW: *P* = 0.045). This heterogeneity may be attributed to genetic interactions, environmental factors, or sample sizes, among other factors. To exclude the impact of heterogeneity, we substituted the fixed-effects IVW with a random-effects IVW model. No heterogeneity was indicated in the remaining six results (*P* > 0.05). After the removal of outliers, the MR-PRESSO results also indicated no horizontal pleiotropy. Furthermore, the Steiger test indicates that there is no reverse causality between MetS and its diagnostic components and CRC (Table 2). LOO analysis confirmed that individual SNPs do not cause bias in MR, and funnel plots demonstrated the stability of the results (Supplementary Figures. S1 and S2).

**Table 2:** In the preliminary analysis, sensitivity analysis of MR results

Exposure	Heterogeneity				Intercept term		MR-PRESSO			Steiger
	MR-Egger		IVW		Intercept	*P*	Global test		Distortion test	*P*
	Q	*P*	Q	*P*			RSSobs	*P*	*P*	
Metabolic syndrome	171.075	0.337	171.136	0.356	0.001	0.810	173.290	0.345		6.26 × 10^–20^
Fasting glucose	31.617	0.169	35.036	0.111	− 0.016	0.113	71.992	< 0.001	0.574	1.27 × 10^–61^
HDL cholesterol	43.627	0.040	44.276	0.045	− 0.006	0.517	45.901	0.066		3.27 × 10^–230^
Triglycerides	18.989	0.458	21.221	0.384	− 0.016	0.152	51.745	0.003	0.707	4.99 × 10^–47^
Systolic blood pressure	203.847	0.218	203.861	0.233	− 0.001	0.909	206.234	0.227		9.69 × 10^–35^
Diastolic blood pressure	203.395	0.285	203.403	0.302	< − 0.001	0.930	273.824	0.016	0.122	3.34 × 10^–33^
Waist circumference	291.094	0.867	291.548	0.871	0.002	0.501	293.679	0.86		2.72 × 10^–52^

*IVW* inverse variance weighted, *RSSobs* residual sum of squares observed

### Replication and meta-analysis

**Fig. 3:**
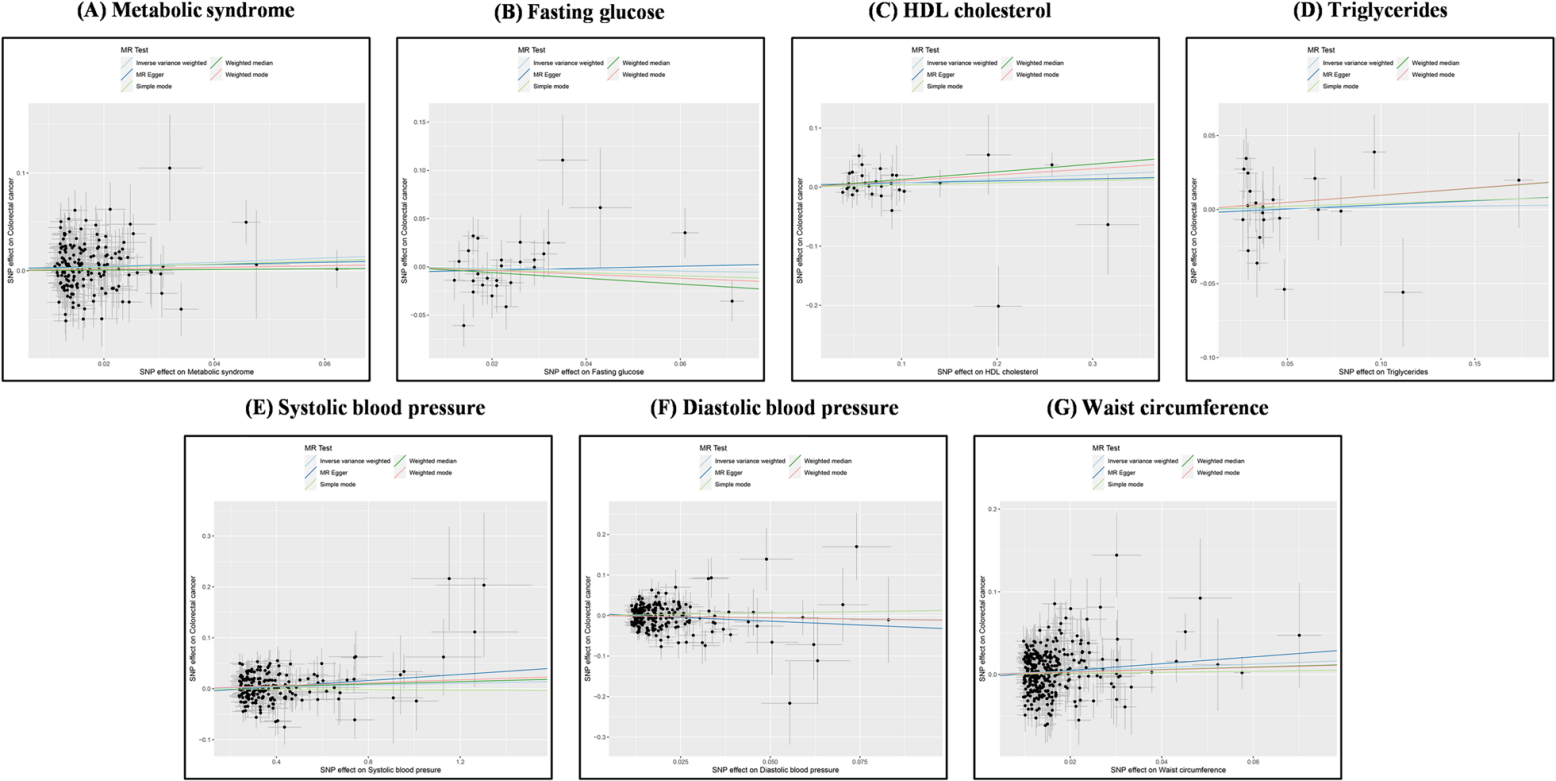
In the replication analysis, scatter plot demonstrated the impact of MetS and its diagnostic components on CRC. **A** Scatter plot of metabolic syndrome and colorectal cancer; **B** Scatter plot of fasting glucose and colorectal cancer; **C** Scatter plot of HDL cholesterol and colorectal cancer; **D** Scatter plot of Triglycerides and colorectal cancer; **E** Scatter plot of systolic blood pressure and colorectal cancer; **F** Scatter plot of diastolic blood pressure and colorectal cancer; **G** Scatter plot of waist circumference and colorectal cancer. *SNP* single nucleotide polymorphism, *MR* Mendelian randomization

**Fig. 4:**
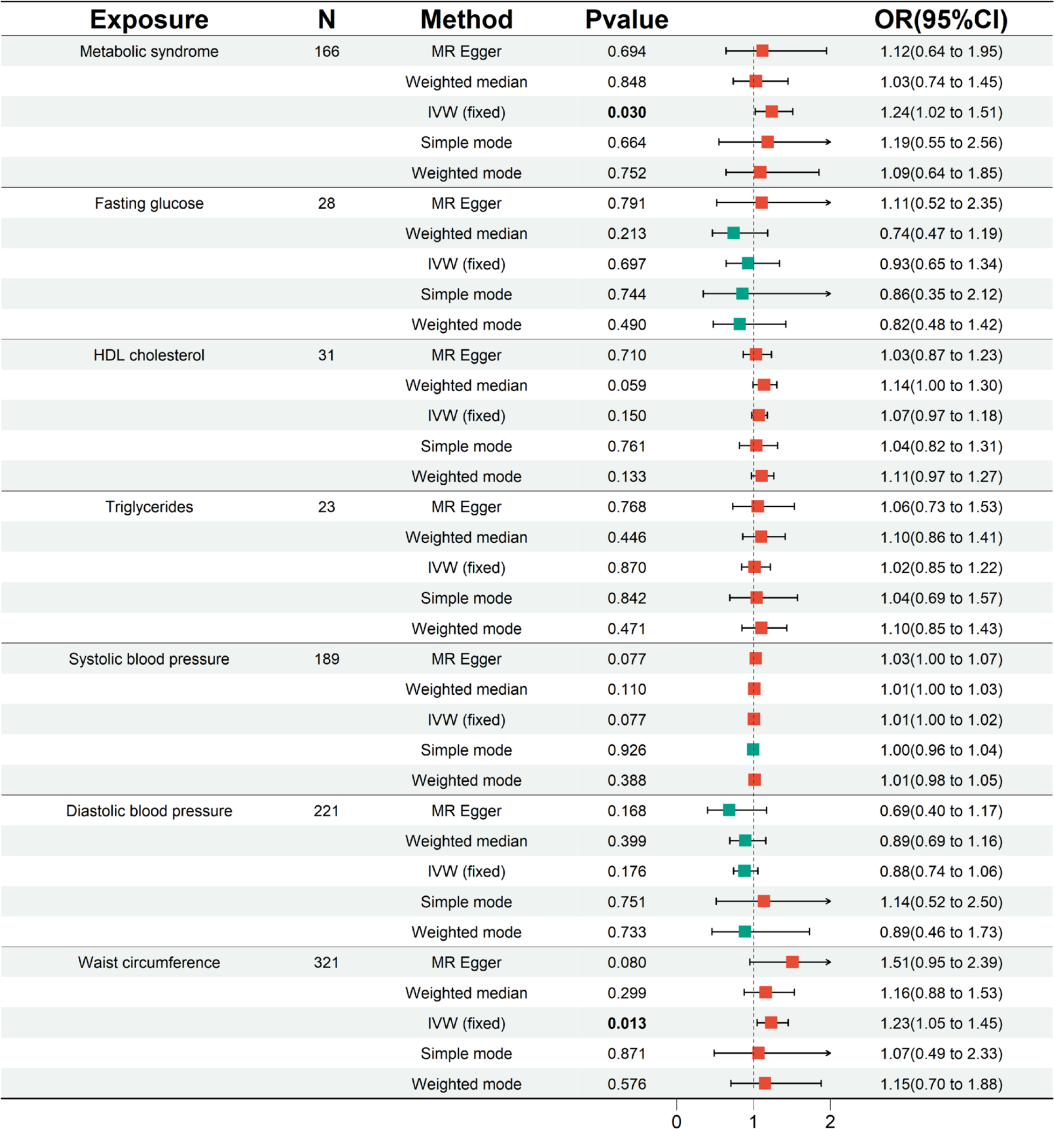
In the replication analysis, forest plot for MetS and its diagnostic components. *95% CI* 95% confidence interval, *OR* odds ratio, *N* the number of SNPs strongly associated with the exposure, *IVW* inverse variance weighted

**Fig. 5:**
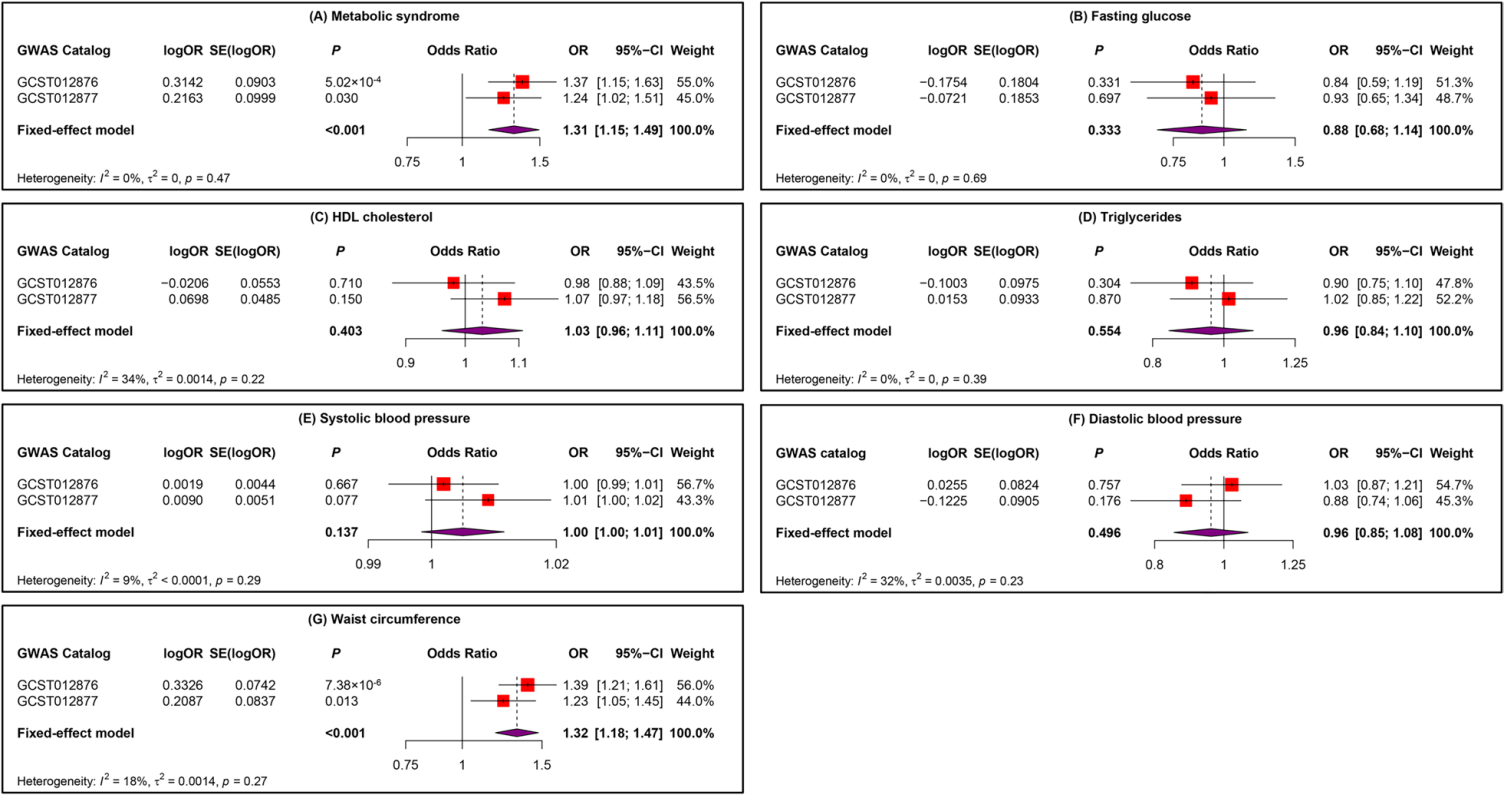
Meta-analyses of the association between MetS and its diagnostic components and CRC. **A** Meta-analysis results of metabolic syndrome; **B** Meta-analysis results of fasting glucose; **C** Meta-analysis results of HDL cholesterol; **D** Meta-analysis results of triglycerides; **E** Meta-analysis results of systolic blood pressure; **F** Meta-analysis results of diastolic blood pressure; **G** Meta-analysis results of waist circumference. *95% CI* 95% confidence interval, *OR* odds ratio, *SE* standard error

To enhance the credibility of our preliminary analysis, we replicated an MR analysis of CRC using an additional GWAS dataset. Initially, we still selected IVs of rigorous quality. In the replication cohort, FG (*P* = 0.008) and DBP (*P* = 0.014) exhibited horizontal pleiotropy, and the MR-PRESSO outlier and distortion test identified and removed all outliers in FG (*P* = 0.891; outliers: rs1260326, rs174576) and DBP (*P* = 0.862; outliers: rs41475048, rs7744284). Ultimately, in the replication cohort, SNPs strongly associated with MetS (N = 166), FG (N = 28), HDL-C (N = 31), TG (N = 23), SBP (N = 189), DBP (N = 221), and WC (N = 321) were selected as genetic IVs. This was followed by an MR replication analysis (Fig. 3 and 4). The IVW results indicated a significant correlation between genetic susceptibility to MetS and increased CRC risk (OR [95% CI] = 1.24[1.02–1.51], *P* = 0.030, statistical power = 81.8%), while an increase in WC was associated with an increased risk of CRC (OR [95% CI] = 1.23[1.05–1.45], *P* = 0.013, statistical power = 89.7%). According to the MR analyses of MetS and WC, the direction and magnitude of the estimates from the five MR methods were consistent (OR > 1). Consistent with the preliminary analysis, no significant causal relationships were detected between the other diagnostic components and CRC (IVW: *P* > 0.05), with statistical power of FG at 12.1%, HDL-C at 34.5%, TG at 4.1%, SBP at 3.4%, and DBP at 39.9%. Additionally, after removing outliers, both the MR‒Egger intercept term and MR-PRESSO results excluded the possibility of horizontal pleiotropy associated with MetS and its diagnostic components, and Cochran’s Q test (*P* > 0.05) also confirmed the absence of heterogeneity between them. The Steiger test also confirmed that there was no reverse causality between MetS and its diagnostic components and CRC (Table 3). LOO analysis and funnel plots confirmed the stability of the results (Supplementary Figures. S3 and S4 online). Finally, we used a meta-analysis of the IVW results from both cohorts (Fig. 5). The results of the meta-analysis further proved that MetS and its diagnostic component WC can influence CRC incidence. Specifically, genetic susceptibility to MetS (OR [95% CI] = 1.31[1.15–1.49], *P* < 0.001) and WC (OR [95% CI] = 1.32[1.18–1.47], *P* < 0.001) increased the risk of CRC. A meta-analysis of the remaining diagnostic components also revealed no causal relationship with CRC (*P* > 0.05). Moreover, Cochran’s Q test demonstrated the absence of heterogeneity (I^2^ < 50% and *P* > 0.05).

**Table 3:** In the replication analysis, sensitivity analysis of MR results

Exposure	Heterogeneity				Intercept term		MR-PRESSO			Steiger
	MR-Egger		IVW		Intercept	*P*	Global test		Distortion test	*P*
	Q	*P*	Q	*P*			RSSobs	*P*	*P*	
Metabolic syndrome	181.975	0.160	182.145	0.171	0.002	0.696	184.372	0.177		6.74 × 10^–15^
Fasting glucose	34.945	0.113	35.312	0.131	− 0.005	0.605	57.283	0.008	0.891	3.47 × 10^–59^
HDL cholesterol	27.894	0.524	28.127	0.567	0.004	0.633	30.548	0.582		5.03 × 10^–224^
Triglycerides	25.000	0.247	25.076	0.293	− 0.002	0.804	27.435	0.323		2.55 × 10^–61^
Systolic blood pressure	218.810	0.055	220.803	0.051	− 0.008	0.193	223.765	0.053		1.26 × 10^–22^
Diastolic blood pressure	250.917	0.068	252.039	0.068	0.005	0.323	278.726	0.014	0.862	5.18 × 10^–24^
Waist circumference	353.901	0.087	354.851	0.087	− 0.003	0.355	360.379	0.074		2.68 × 10^–33^

*IVW* inverse variance weighted, *RSSobs* residual sum of squares observed

## Discussion

In our study, we integrated two large-scale, publicly available GWAS datasets related to CRC. Through rigorous MR analysis, we explored the impact of genetic susceptibility to MetS and its diagnostic components on CRC incidence. We conducted a meta-analysis of the IVW results from both the preliminary and replication analyses. Ultimately, we established a significant correlation between genetically determined MetS and WC and an increased risk of CRC.

Epidemiological evidence has demonstrated a markedly elevated risk of CRC in individuals diagnosed with MetS [32]. A recent meta-analysis concluded that there is a positive correlation between MetS and the risk of CRC [33], aligning with the findings of our study. However, the association between MetS and CRC incidence remains controversial due to inconsistent findings in previous research, such as gender differences. A study by Pelucchi et al. [34], revealed that MetS increased the risk of CRC in males but not in females. This gender disparity may be attributed to differences in sex hormone levels between males and females. It has been suggested that estrogen can reduce the risk of CRC by decreasing the synthesis and secretion of bile acids and exerting anticancer effects through the inhibition of cell proliferation and promotion of apoptosis [35–37]. Nevertheless, recent studies have indicated that MetS is associated with an increased incidence and mortality rate of CRC in both males and females [38]. We posit that the observed discrepancies might stem from the influence of confounding factors characteristic of observational study designs. Notably, MR analysis, which uses genetic variants as IVs randomly assigned at conception, significantly minimizes the impact of confounding factors. Therefore, our study substantially reduces these influences.

Furthermore, among the diagnostic components of MetS, an increase in WC significantly elevates the incidence of CRC. WC is a reliable indicator for identifying abdominal obesity and a core criterion for diagnosing MetS, as it is positively correlated with visceral adipose tissue volume [39]. Abdominal obesity is closely linked to CRC. Abdominal obesity can increase the risk of developing CRC, with accumulations of subcutaneous and visceral fat significantly increases the mortality risk in CRC patients [40, 41]. This difference may be associated with the secretion of adipokines such as leptin and resistin by adipose tissue. Many studies indicate that the overexpression of leptin correlates with later stages of CRC. Leptin not only regulates angiogenesis and cell apoptosis through various pathways but also activates the production of inflammatory cytokines such as IL-6 and TNF-α, thereby promoting CRC development [42]. Resistin can act as a risk factor and potential prognostic biomarker in CRC. It has been shown that resistin activates macrophages involved in inflammatory processes and stimulates angiogenesis through various proangiogenic molecules, including VEGF receptors, ultimately promoting the growth and metastasis of CRC [43].

In contrast, our study did not find significant causal relationships between other diagnostic components of MetS besides WC and the risk of CRC. Although the current study results show a lack of significant association, our calculation of statistical power revealed that, except for WC, the statistical power of other diagnostic components was below 80%. This suggests that even if there is indeed a causal relationship between them and CRC, this study may not be able to effectively detect it. Several studies suggest that insulin resistance is a primary mechanism of MetS. Elevated levels of insulin in the body, through binding with cell surface IGF-1 receptors, promote cell proliferation, thereby facilitating the development of CRC [44]. Epidemiological studies indicated that diabetes is a risk factor for CRC, particularly type 2 diabetes. A MR study by Murphy et al., shows that high fasting insulin levels are associated with an increased risk of CRC, rather than high FG levels or type 2 diabetes [45]. However, obesity is widely considered one of the significant factors contributing to insulin resistance, especially abdominal obesity. Research by Tian et al. [46], indicated that high levels of total serum cholesterol, TG, and HDL-C are positively correlated with colorectal adenomas, which are precancerous lesions of CRC with a prolonged risk of developing CRC. Fang et al. [47], after adjusting for potential confounders such as BMI and WC, found no correlation between dyslipidemia and CRC risk. Currently, the relationship between hypertension and CRC remains unclear, and reports on this topic are limited. One study comparing early-onset CRC patients with healthy controls revealed a significant correlation between hypertension, as one of the complications of obesity, and increased CRC risk [48]. Although some observational studies have suggested links between FG, SBP, DBP, TG, and HDL-C and CRC incidence, we believed that these associations may be mediated by abdominal obesity.

This study is subject to certain limitations. Firstly, due to the relative scarcity of GWAS datasets related to the exposure and outcome factors in our study, we conducted MR analysis using GWAS data from individuals of European ancestry only. However, in different ethnic groups, the same genetic variants may exhibit different magnitudes or directions of effects due to diverse genetic backgrounds. Therefore, this specificity restricts the extrapolation of our findings to broader, more diverse populations. Secondly, as previously mentioned, due to the lack of data, the GWAS datasets we used are not sufficient to address the issue of inadequate statistical power, thus certain results should be interpreted with caution. To improve the statistical power of the study, future research should consider using larger sample sizes or more effective genetic instrumental variables to accurately estimate causal relationships. Moreover, although our GWAS data included both male and female samples, which significantly reduced the impact of confounding factors, we cannot fully elucidate whether gender differences affect the causal relationship between MetS and CRC. In light of this, we believe that further gender-stratified research is necessary. Unfortunately, we were unable to obtain genetic information specific to male and female MetS patients. Finally, our study exhibits some degree of survivor bias. Due to the late onset of CRC, publicly released GWAS datasets may overlook individuals who died or failed to be diagnosed before the development of the disease under study, leading to biased estimates of the genetic associations involved. Meta-analysis can provide more stable estimates by pooling data and potentially eliminating individual biases, which somewhat mitigated the impact of survivor bias. But it is noteworthy that we must still carefully consider the characteristics of each dataset, such as the variability of different GWAS datasets, the diversity of the ancestries of the individuals studied, and the late onset of CRC, to effectively comprehend survivor bias.

## Conclusion

In conclusion, this study provides reliable evidence supporting a causal relationship between MetS and CRC. In view of this, clinicians should be particularly attentive to the potential risk of CRC in patients with MetS. This approach is especially pertinent for patients with larger waist circumferences who are obese, where an emphasis on enhanced CRC screening and preventive measures is essential.

### Supplementary Information

Supplementary Material 1.

Supplementary Material 2: Figure S1. In preliminary analysis, using the leave-one-out analysis as a genetically determined approach to identify the sensitivity to colorectal cancer influenced by the metabolic syndrome and its diagnostic components. Figure S2. In preliminary analysis, demonstrating the stability of causal relationship results of the metabolic syndrome and its diagnostic components on CRC through a funnel plot. Figure S3: In replication analysis, using the leave-one-out analysis as a genetically determined approach to identify the sensitivity to colorectal cancer influenced by the metabolic syndrome and its diagnostic components. Figure S4: In replication analysis, demonstrating the stability of causal relationship results of the metabolic syndrome and its diagnostic components on CRC through a funnel plot.

## Data Availability

In the Materials and methods section of our article, we have indicated the sources of all original data. If needed, please contact the original authors for access. The results of this study can be obtained by contacting the corresponding author.

## Abbreviations

CRC: Colorectal cancer
MetS: Metabolic syndrome
WC: Waist circumference
DBP: High diastolic blood pressure
SBP: Systolic blood pressure
TG: Triglyceride
HDL-C: HDL cholesterol
FG: Fasting glucose
SNPs: Single nucleotide polymorphisms
IVs: Instrumental variables
GWAS: Genome-wide association study
IVs: Instrumental variables
MR: Mendelian randomization
IVW: Inverse variance weighted
LOO analysis: Leave-one-out analysis
OR: Odds ratio
CI: Confidence interval
PVE: Proportion of variance explained
