# Supplementary figures and images for: Genetic prediction of the relationship between metabolic syndrome and colorectal cancer risk: a Mendelian randomization study
Source: Diabetol Metab Syndr. 2024 May 22;16:109. doi: 10.1186/s13098-024-01351-7 (PMC11110320; doi:10.1186/s13098-024-01351-7)

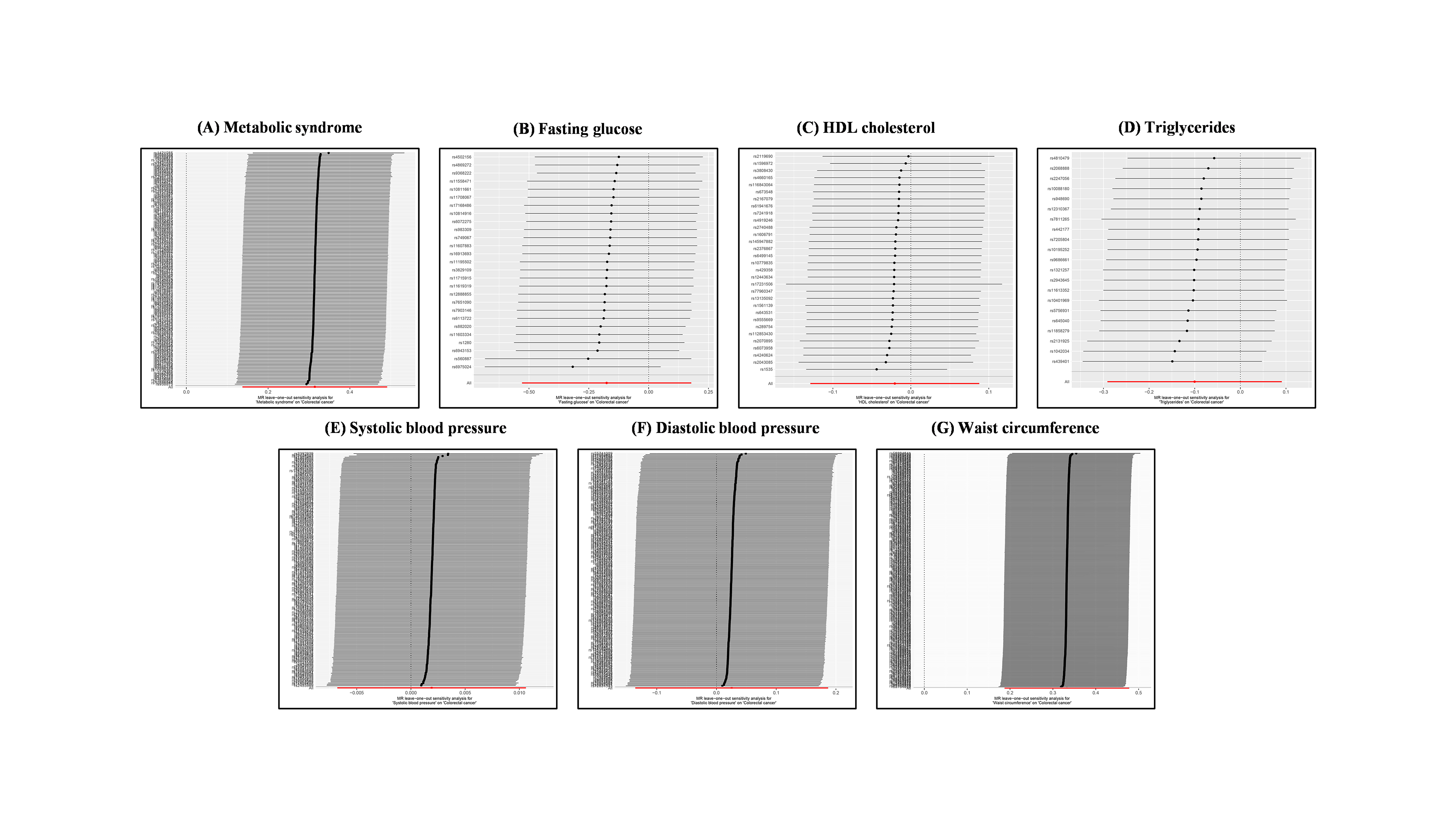

Supplement: Supplementary file 2 — Supplementary Material 2: Figure S1. In preliminary analysis, using the leave-one-out analysis as a genetically determined approach to identify the sensitivity to colorectal cancer influenced by the metabolic syndrome and its diagnostic components. Figure S2. In preliminary analysis, demonstrating the stability of causal relationship results of the metabolic syndrome and its diagnostic components on CRC through a funnel plot. Figure S3: In replication analysis, using the leave-one-out analysis as a genetically determined approach to identify the sensitivity to colorectal cancer influenced by the metabolic syndrome and its diagnostic components. Figure S4: In replication analysis, demonstrating the stability of causal relationship results of the metabolic syndrome and its diagnostic components on CRC through a funnel plot. [file 13098_2024_1351_MOESM2_ESM.zip › New folder/Supplementary Figure. S1.tif]

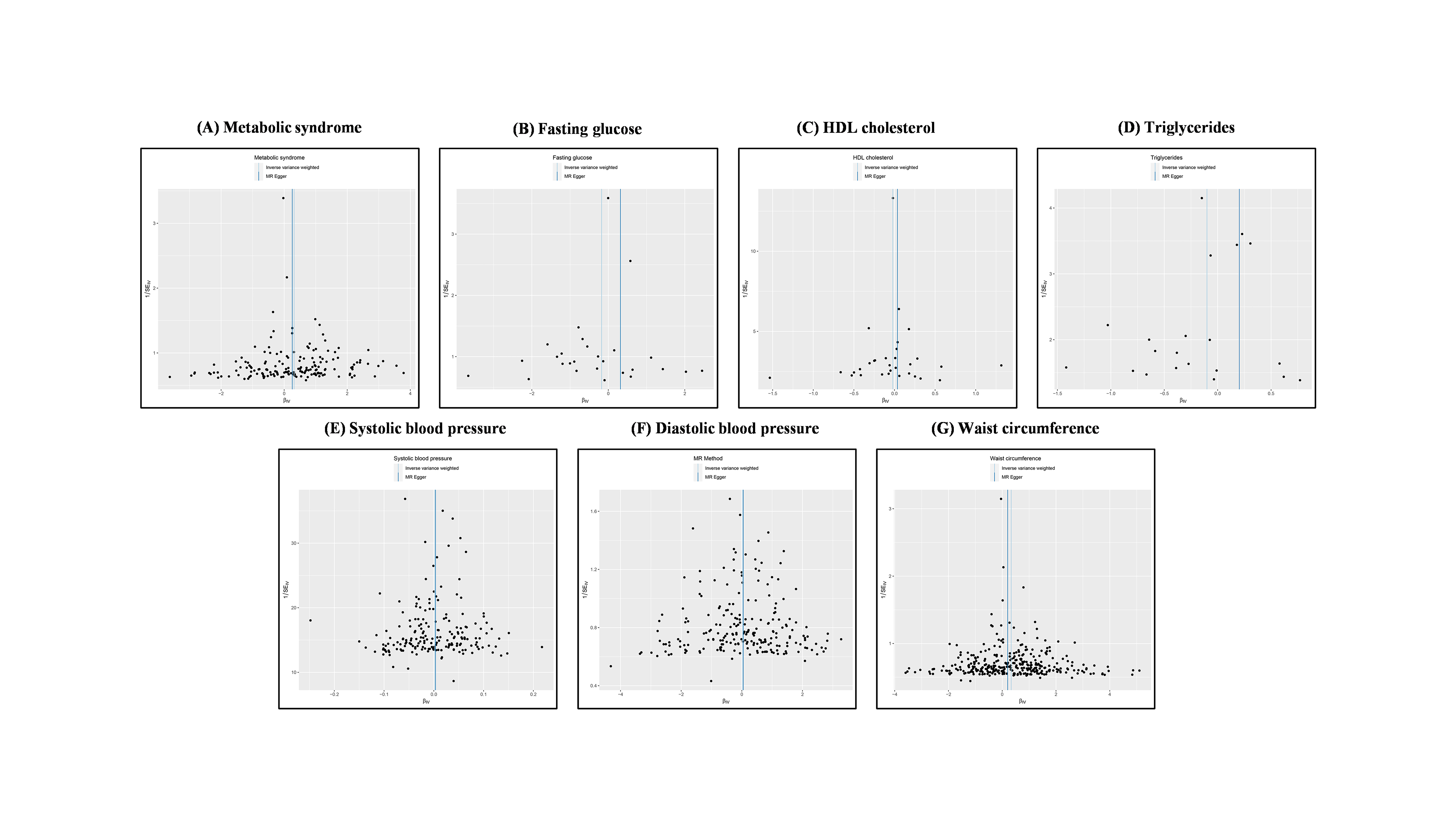

Supplement: Supplementary file 2 — Supplementary Material 2: Figure S1. In preliminary analysis, using the leave-one-out analysis as a genetically determined approach to identify the sensitivity to colorectal cancer influenced by the metabolic syndrome and its diagnostic components. Figure S2. In preliminary analysis, demonstrating the stability of causal relationship results of the metabolic syndrome and its diagnostic components on CRC through a funnel plot. Figure S3: In replication analysis, using the leave-one-out analysis as a genetically determined approach to identify the sensitivity to colorectal cancer influenced by the metabolic syndrome and its diagnostic components. Figure S4: In replication analysis, demonstrating the stability of causal relationship results of the metabolic syndrome and its diagnostic components on CRC through a funnel plot. [file 13098_2024_1351_MOESM2_ESM.zip › New folder/Supplementary Figure. S2.tif]

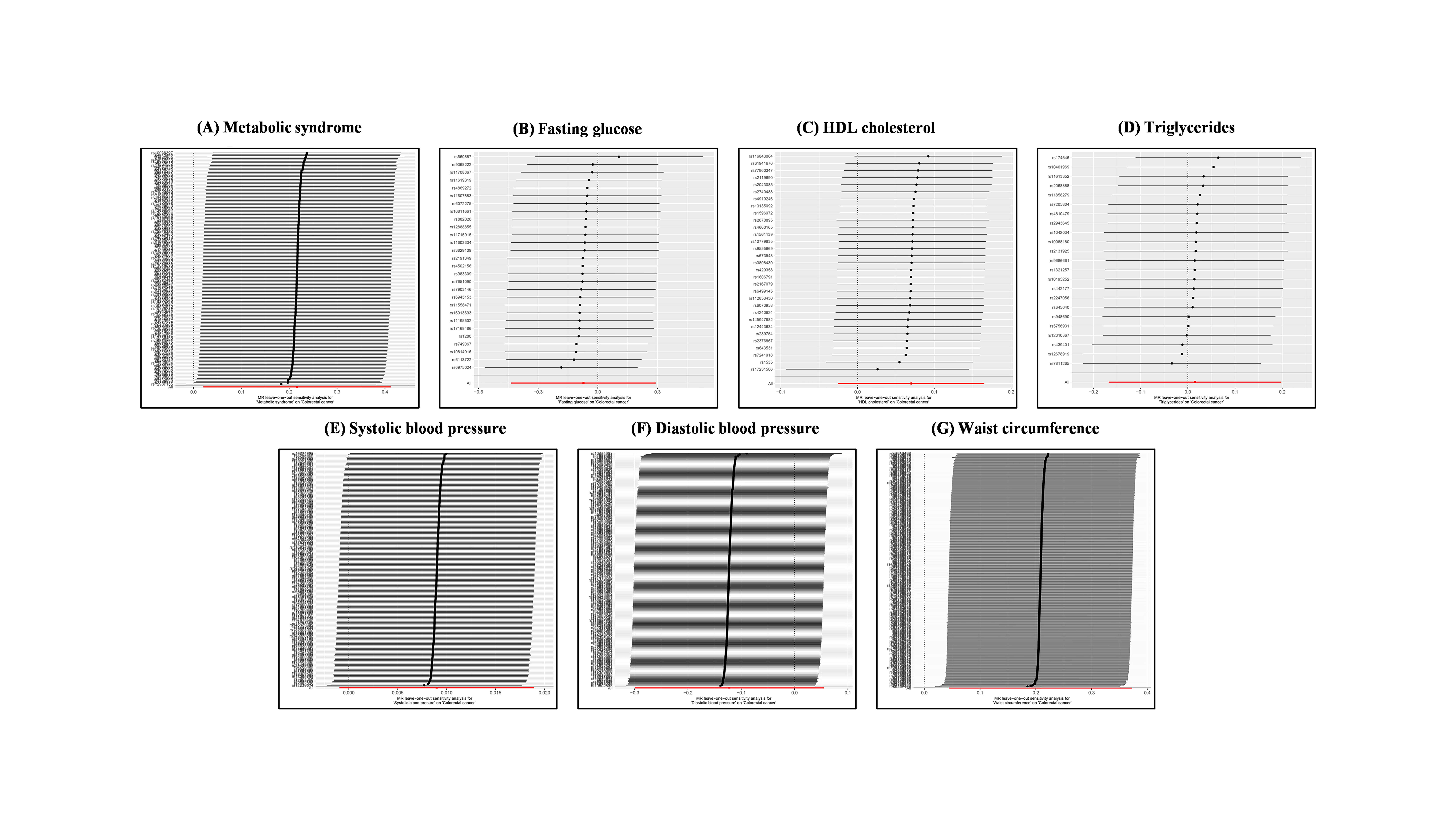

Supplement: Supplementary file 2 — Supplementary Material 2: Figure S1. In preliminary analysis, using the leave-one-out analysis as a genetically determined approach to identify the sensitivity to colorectal cancer influenced by the metabolic syndrome and its diagnostic components. Figure S2. In preliminary analysis, demonstrating the stability of causal relationship results of the metabolic syndrome and its diagnostic components on CRC through a funnel plot. Figure S3: In replication analysis, using the leave-one-out analysis as a genetically determined approach to identify the sensitivity to colorectal cancer influenced by the metabolic syndrome and its diagnostic components. Figure S4: In replication analysis, demonstrating the stability of causal relationship results of the metabolic syndrome and its diagnostic components on CRC through a funnel plot. [file 13098_2024_1351_MOESM2_ESM.zip › New folder/Supplementary Figure. S3.tif]

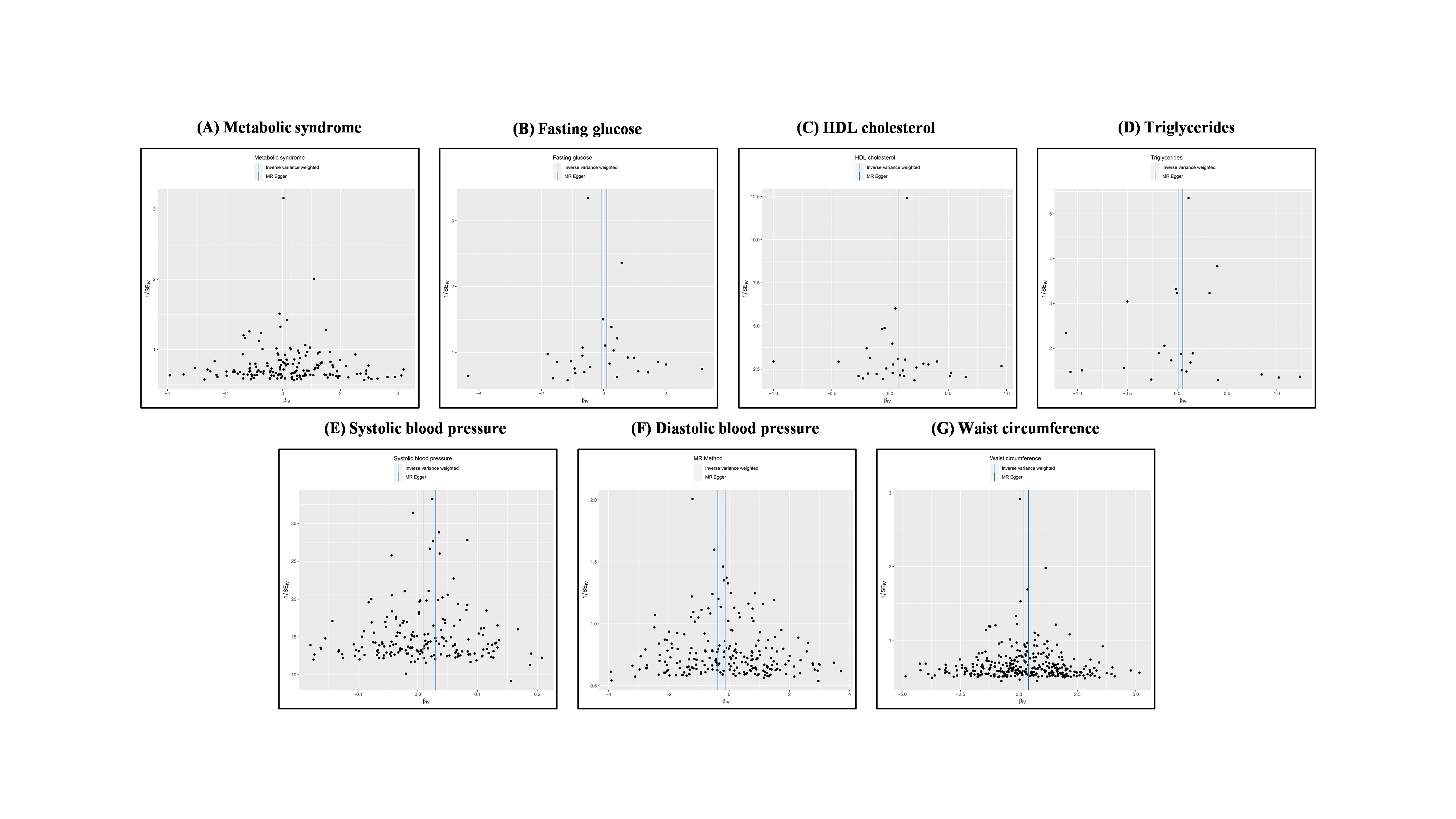

Supplement: Supplementary file 2 — Supplementary Material 2: Figure S1. In preliminary analysis, using the leave-one-out analysis as a genetically determined approach to identify the sensitivity to colorectal cancer influenced by the metabolic syndrome and its diagnostic components. Figure S2. In preliminary analysis, demonstrating the stability of causal relationship results of the metabolic syndrome and its diagnostic components on CRC through a funnel plot. Figure S3: In replication analysis, using the leave-one-out analysis as a genetically determined approach to identify the sensitivity to colorectal cancer influenced by the metabolic syndrome and its diagnostic components. Figure S4: In replication analysis, demonstrating the stability of causal relationship results of the metabolic syndrome and its diagnostic components on CRC through a funnel plot. [file 13098_2024_1351_MOESM2_ESM.zip › New folder/Supplementary Figure. S4.tif]
